# Safety and Clinical Performance of Peripherics™ Paclitaxel-Coated Balloon Catheter in Below-the-Knee Arteries: A Multicenter Post-market Study

**DOI:** 10.7759/cureus.112622

**Published:** 2026-07-13

**Authors:** Prashant Sarda, PremChand Gupta, Pradeep Burli, Luv Luthra, Varinder Bedi

**Affiliations:** 1 Vascular Surgery, Shri Mahant Indiresh Hospital, Dehradun, IND; 2 Vascular Surgery, CARE Hospital, Hyderabad, IND; 3 Vascular Surgery, Global Heart and Super Speciality Hospital, Ludhiana, IND; 4 Vascular Surgery, Institute of Vascular and Endovascular Sciences, Sir Ganga Ram Hospital, New Delhi, IND

**Keywords:** angioplasty, balloon, endovascular procedures, limb-threatening ischemia, paclitaxel, peripheral arterial disease

## Abstract

Introduction: Below-the-knee (BTK) arterial disease is associated with a high risk of limb loss, yet evidence supporting the use of paclitaxel-coated balloons (PCB) in routine clinical practice remains limited.

Aim: This study aimed to evaluate the safety and clinical performance of Peripherics^™^ PCB catheter (Sahajanand Medical Technologies Limited, Surat, India) in the treatment of BTK arteries during routine clinical practice.

Methods: This retrospective, observational, multicenter, single-arm, investigator-initiated, post-market study was conducted between September 2022 and September 2024 at four centers in India. All consecutive patients aged ≥18 years with Rutherford categories III-V, having de novo or non-stented restenotic lesions in one or two native arteries (below the tibial plateau and above the tibiotalar joint), and treated with at least one Peripherics^™^ PCB catheter were included. The primary safety endpoint was 30-day major adverse limb events (MALE) and perioperative all-cause death (POD). MALE comprised above-ankle amputation or major reintervention involving a BTK artery.

Results: A total of 71 Peripherics^™^ PCB catheters were used in 70 patients (mean age: 63.3±11.9 years; n=58; 82.9% male) to treat 70 lesions in BTK arteries. Rutherford class V was observed in the majority of patients (n=44; 62.9%). Thrombus was present in 39 (55.7%) lesions, total occlusion in 25 (35.7%), and calcification in 28 (40%) lesions. Device success, technical success, and procedural success were 100% (n=71), 100% (n=70), and 97.1% (n=68), respectively. The mean ankle-brachial index improved from 0.51±0.15 at baseline to 0.79±0.16 at 30 days. At 30 days, the MALE with POD was observed in two (2.9%) patients, including one patient who required thrombolysis followed by major reintervention (1.4%) and one perioperative death (1.4%). Clinical improvement was evident in 60 (87%) patients who demonstrated improvement in at least one Rutherford class.

Conclusion: The Peripherics^™^ PCB catheter demonstrated favorable safety and clinical performance in the treatment of BTK arteries, with low event rates and marked clinical improvement at 30 days.

## Introduction

Below-the-knee (BTK) arterial disease can lead to critical limb ischemia characterized by rest pain, non-healing ulcers, and tissue loss with a high risk of amputation and mortality [1,2]. The burden is especially high in low- and middle-income countries such as India, where patients often present late with advanced ischemia, multilevel disease, and multiple coexisting cardiovascular risk factors [3]. Endovascular therapy is preferred over surgical bypass for revascularization in patients with BTK disease because of its lower procedural risk and broader applicability in routine practice [4]. However, restenosis remains a significant limitation following plain balloon angioplasty in infrapopliteal vessels, driven by small vessel caliber, diffuse calcification, recoil, and aggressive neointimal hyperplasia [5]. Paclitaxel-coated balloons (PCB) provide local antiproliferative drug delivery without permanent implant placement, which is particularly advantageous in BTK arterial disease, where stent durability is limited and long diffuse disease is frequent [6].

The Peripherics™ PCB catheter (Sahajanand Medical Technologies Limited, Surat, India) delivers paclitaxel at a uniform surface dose density of 2 μg/mm² along the balloon working length. The catheter is designed to achieve optimal drug transfer while maintaining procedural compatibility with standard over-the-wire BTK angioplasty techniques. This characteristic may be particularly relevant in BTK lesions, in which early drug availability and sufficient tissue drug retention are key determinants of restenosis prevention. Although PCB have demonstrated favorable outcomes in femoropopliteal disease, real-world evidence supporting their use in BTK arterial disease remains limited.

Accordingly, this study was designed to evaluate the safety and clinical performance of Peripherics™ PCB catheter in the treatment of BTK arteries during routine clinical practice in Indian patients.

## Materials and methods

Study design and population

This retrospective, observational, multicenter, single-arm, investigator-initiated, post-market study was conducted at four centers in India from September 2022 through September 2024: Shri Mahant Indiresh Hospital, Dehradun; CARE Hospital, Banjara Hills, Hyderabad; Global Heart and Super Speciality Hospital, Ludhiana; and Sir Ganga Ram Hospital, New Delhi.** **We included consecutive patients who had undergone treatment with the Peripherics™ PCB catheter for BTK arterial disease. Patients who had been treated with the study device within six months before or after the initiation of retrospective anonymous data collection were enrolled. Eligible patients were ≥18 years of age (male or non-pregnant, non-breastfeeding female) and presented with peripheral arterial disease manifesting as claudication or critical limb ischemia corresponding to Rutherford categories III-V. The target lesions were significant stenosis or occlusions in one or two native infrapopliteal arteries located between the tibial plateau and the tibiotalar joint, appropriate for angioplasty per the operator's visual assessment. Lesions were either de novo or non-stented restenotic; any prior angioplasty of the target lesion had to have been performed more than 30 days before the index procedure. In all cases, at least one Peripherics™ PCB catheter was used.

Patients were excluded if they had a life expectancy of less than one year, had lesions extending into the popliteal artery or below the ankle joint, or had significant inflow disease involving the ipsilateral iliac, superficial femoral, or popliteal artery with a lesion length of ≥15 cm. Additional exclusion criteria included the following: (1) concurrent participation in another investigational drug or device trial that has not reached its primary endpoint yet, (2) any medical condition that would make the patient unsuitable for treatment with Peripherics™ PCB catheter in the opinion of the investigator, (3) planned major amputation above the ankle of the target limb or any other major surgery within 30 days of the procedure, (4) prior stent implantation in the target lesion, or (5) treatment with a drug-coated balloon (DCB) in the target vessel within six months before the index procedure.

The study protocol was approved by the Ethics Committee of Sir Ganga Ram Hospital (approval number: EC/10/23/2381) under a lead principal investigator model. Participating centers contributed anonymized retrospective data under the same approved study protocol. As this was a retrospective analysis, the requirement for informed consent was waived. This study was conducted in accordance with the Declaration of Helsinki and applicable regulatory requirements.

Study device

The Peripherics™ PCB catheter is an over-the-wire percutaneous transluminal angioplasty (PTA) device with a PCB fixed at the distal tip. The device is designed to deliver a controlled paclitaxel dose to the vessel wall during balloon inflation. The paclitaxel coating is evenly distributed across the working length of the balloon at a surface concentration of 2 ug/mm^2^ providing an antiproliferative effect to reduce restenosis. The device is available in balloon diameters ranging from 2 to 12 mm and lengths ranging from 20 to 200 mm, with the corresponding paclitaxel dosage varying according to the balloon dimensions. The catheter incorporates a dual-lumen shaft that branches into two ports at the proximal end: one for guidewire access and the other for balloon inflation and deflation with a mixture of contrast medium and saline solution. The balloon is equipped with two radiopaque marker bands that define the working length and facilitate precise positioning during delivery and inflation under fluoroscopic guidance.

Data collection

All relevant clinical and procedural data were extracted from patient medical records and hospital databases for this retrospective analysis. Patient demographics, comorbidities, Rutherford classification [7], Trans-Atlantic Inter-Society Consensus (TASC) II classification [4], ankle-brachial index (ABI) index [8], procedural details, device specifications, and periprocedural complications were collected. Procedural and imaging records were reviewed to confirm the location, severity, and treatment approach of the lesion. Thrombus burden was assessed angiographically during the index procedure based on operator evaluation. Use of thrombolytic therapy was left to the physician's discretion according to lesion characteristics, thrombus burden, distal flow compromise, and institutional practice. The index procedure was defined as the treatment of the target BTK lesion with at least one Peripherics™ PCB catheter.

Endpoints and follow-up

The primary safety endpoint was major adverse limb events (MALE) with perioperative all-cause death (POD) at 30 days. MALE was defined as a composite of above-ankle amputation or major reintervention, including new bypass graft, jump/interposition graft revision, or thrombectomy/thrombolysis of the treated limb involving a BTK artery. POD was defined as all-cause death within 30 days after the index procedure.

Secondary endpoints included major adverse events (MAE) at 30 days, two months, three months, and six months, defined as a composite of all-cause mortality, major amputation of the treated limb, and clinically driven target lesion revascularization (CD-TLR), defined as any reintervention performed for ≥50% diameter stenosis (visual estimate) at the target lesion after the documentation of recurrent clinical symptoms. Additional secondary endpoints were primary patency at six months, defined as freedom from CD-TLR and restenosis on duplex ultrasound; all-cause mortality; major amputation; CD-TLR; improvement in Rutherford class; amputation-free survival (AFS) including major, minor, and overall AFS; and changes in mean ABI or transcutaneous oxygen pressure (TcPO₂), all assessed at 30 days, two months, three months, and six months.

Procedural endpoints included device success, defined as successful delivery, inflation, deflation, and retrieval of the device; technical success, defined as successful completion of the endovascular procedure with immediate morphological success and ≤50% residual diameter reduction; and procedural success, defined as achievement of both device and technical success without any MAE during the hospital stay. The present analysis reports 30-day outcomes and will continue to be a routine hospital follow-up for two, three, and six months.

Statistical analysis

All continuous measures are presented as means±standard deviations (SD). Categorical variables are presented as numbers (n) and percentages (%). The primary endpoints will be analyzed using the Kaplan-Meier method based on the time-to-event survival analysis. All data were analyzed using R Version 4.3.3. (The R Foundation for Statistical Computing, Vienna, Austria).

## Results

Baseline demographic and clinical characteristics

A total of 70 patients with BTK arterial disease and treated with Peripherics™ PCB catheter were included. The mean age was 63.3±11.9 years, and the majority were male (n=58; 82.9%). A history of peripheral artery disease was present in 50 (71.4%) patients, while prior peripheral interventions or surgeries were performed in 25 (35.7%). Most patients presented with advanced limb ischemia, with 44 patients (62.9%) classified as Rutherford class V. The mean ABI at baseline was 0.51±0.15. Table 1 describes the baseline demographic and clinical characteristics of the participants.

**Table 1:** Baseline demographics and clinical characteristics of the patients Data are represented as mean±SD or n (%). ABI: ankle-branchial index; BP: blood pressure; COPD: chronic obstructive pulmonary disease; TcPO₂: transcutaneous oxygen pressure; TIA: transient ischemic attack; TP: toe pressure

Variables	Patients (N=70)
Age, years	63.30±11.90
Male	58 (82.9)
Height, cm	167.37±7.64
Weight, kg	69.49±7.32
Heart rate, bpm	80.84±14.56
Systolic BP, mmHg	129.60±14.06
Diastolic BP, mmHg	70.81±12.80
Hypertension	25 (35.7)
Dyslipidemia	24 (34.3)
Diabetes mellitus	28 (40)
Non-insulin dependent	21 (75)
Insulin dependent	7 (25)
Active smoking	7 (10)
Previous smoking	39 (55.7)
Previous coronary artery disease	24 (34.3)
History of peripheral artery disease	50 (71.4)
Previous peripheral interventions/surgeries	25 (35.7)
Previous cerebrovascular disease	8 (11.4)
Renal insufficiency	3 (4.3)
Prior stroke/TIA	9 (12.9)
Previous amputation	16 (22.9)
Minor	15 (93.8)
Major	1 (6.2)
COPD	2 (2.9)
Cancer or malignancy	0 (0)
Rutherford class	
III	8 (11.4)
IV	18 (25.7)
V	44 (62.9)
ABI	0.51±0.15
TP or TcPO₂ measured	19 (27.1)
<30 mmHg	13/19 (68.4)
30-39 mmHg	6/19 (31.6)

Lesion characteristics, procedural features, and angiographic outcomes

The left limb was treated in 52.9% (n=37) of cases, and 52.9% (n=33) of patients had multivessel involvement. Most lesions were de novo (n=65; 92.9%) and focal (n=44; 62.9%). Total occlusions were observed in 25 (35.7%), and thrombus was present in 39 (55.7%). According to TASC II classification, most lesions were B or C (n=24 (34.3%) and n=22 (31.4%), respectively). Pre-dilation was performed in 94.3% (n=66) of cases at a mean inflation pressure of 7.96±1.62 atm. A total of 71 Peripherics™ PCB catheters were used, with a mean diameter of 2.62±0.43 mm and a mean length of 126.48±20.43 mm. Post-dilation was required in 12.9% (n=9) of procedures. Perfusion of artery flow 3 was achieved in 94.3% (n=66) of cases post-procedure. Procedural complications were infrequent and included flow-limiting dissection in 2.8% (n=2), thrombosis in 1.4% (n=1), and post-procedure occlusion in 1.4% (n=1) of patients. No cases of arterial perforation, distal embolization, access site hematoma, or bleeding were recorded. Device success (n=71; 100%), technical success (n=70; 100%), and procedural success (n=68; 97.1%) were consistently high. Details of lesion characteristics, procedural details, and angiographic outcomes are provided in Table 2.

**Table 2:** Lesion characteristics, procedural details, and angiographic outcomes Data are represented as mean±SD or n (%). DCB: drug-coated balloon; TASC II: Trans-Atlantic Inter-Society Consensus II

Variables	Patients (N=70)
Target limb	
Left limb	37 (52.9)
Right limb	33 (47.1)
Extent of disease	
Single-vessel disease	33 (47.1)
Multiple-vessel disease	37 (52.9)
Target vessel	
Distal popliteal artery	0 (0)
Anterior tibial artery	30 (42.9)
Posterior tibial artery	36 (51.4)
Tibioperoneal trunk	4 (5.7)
Peroneal artery	0 (0)
De novo	65 (92.9)
Restenosis	5 (7.1)
Lesion morphology	
Focal	44 (62.9)
Diffuse	26 (37.1)
Thrombus present	39 (55.7)
Total occlusion	25 (35.7)
Calcification	28 (40)
Mild	7 (25)
Moderate	18 (64.3)
Heavy	3 (10.7)
TASC II classification	
A	18 (25.7)
B	24 (34.3)
C	22 (31.4)
D	6 (8.6)
Flow of perfusion of the artery, pre-procedure	
0	21 (30)
1	32 (45.7)
2	17 (24.3)
Thrombolytic therapy	29 (41.4)
Pre-dilation	66 (94.3)
Maximum pressure, atm	7.96±1.62
Access sheath size, Fr	5.83±0.42
Total number of DCB	71
DCB diameter, mm	2.62±0.43
DCB length, mm	126.48±20.43
Post-dilation	9 (12.9)
Maximum pressure, atm	7.06±0.24
Cumulative inflation time, sec	141.18±33.86
Flow of perfusion of the artery, post-procedure	
1	1 (1.4)
2	3 (4.3)
3	66 (94.3)
Procedural complication	
Flow-limiting dissection	2 (2.8)
Thrombosis	1 (1.4)
Post-procedure occlusion	1 (1.4)
Arterial perforation	0 (0)
Access site hematoma	0 (0)
Retroperitoneal hematoma	0 (0)
Distal embolization	0 (0)
Bleeding	0 (0)
Acute renal failure	0 (0)
Emergency surgery	0 (0)
Bailout stenting	0 (0)
Myocardial infarction	0 (0)
Vascular spasm	0 (0)
Device success	71 (100)
Technical success	70 (100)
Procedure success	68 (97.1)

Clinical outcomes

**Figure 1:**
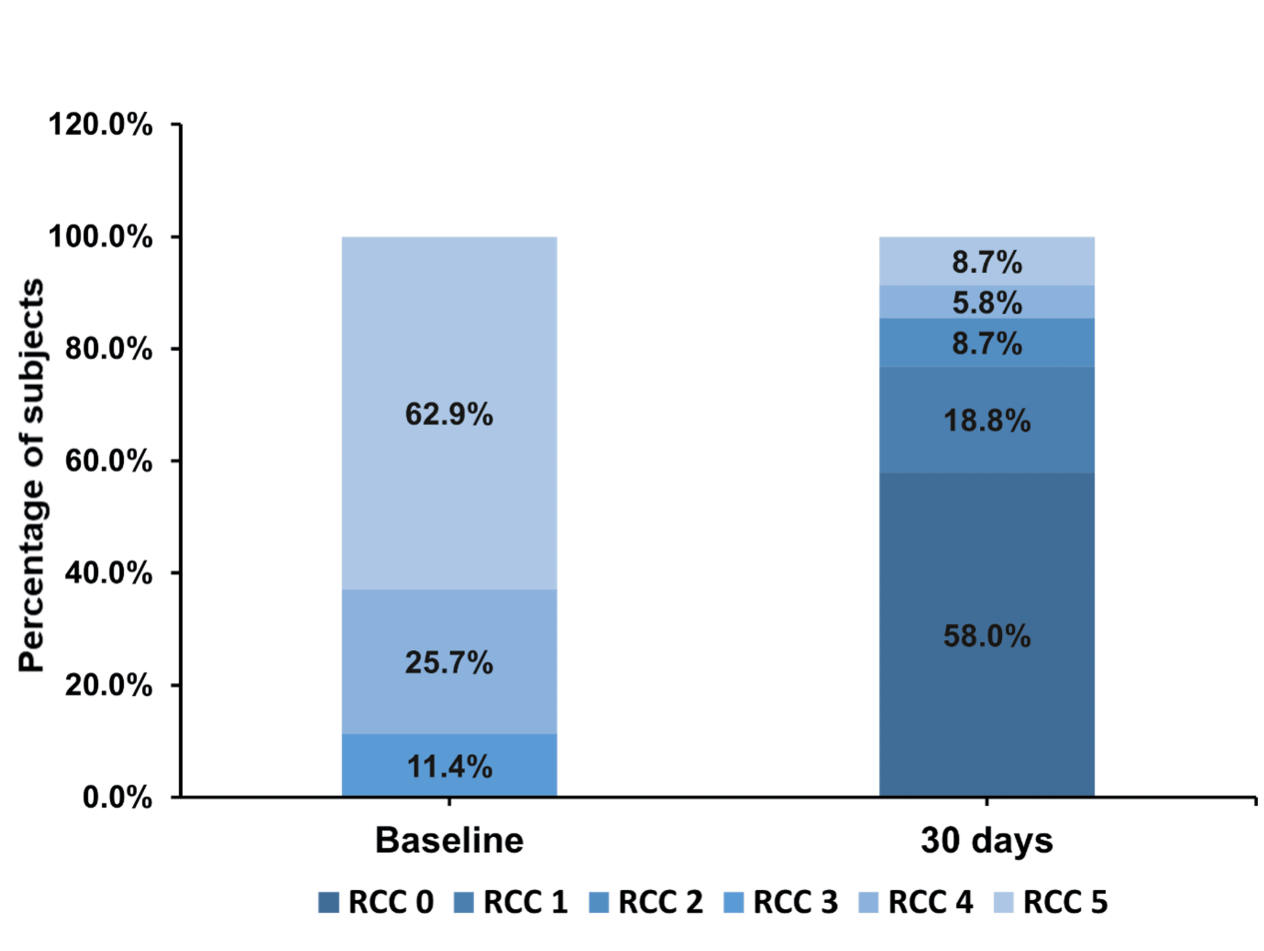
Changes in Rutherford classification from baseline to 30 days post-procedure RCC: Rutherford clinical category

**Figure 2:**
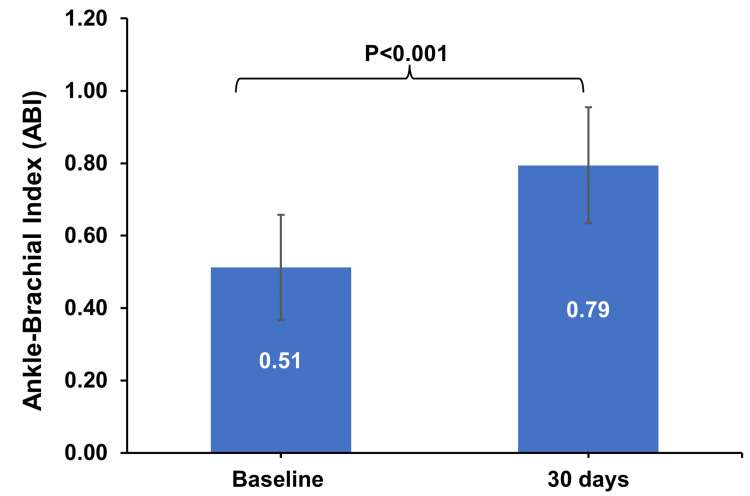
Comparison of ankle-brachial index values before the procedure and at the 30-day follow-up

**Figure 3:**
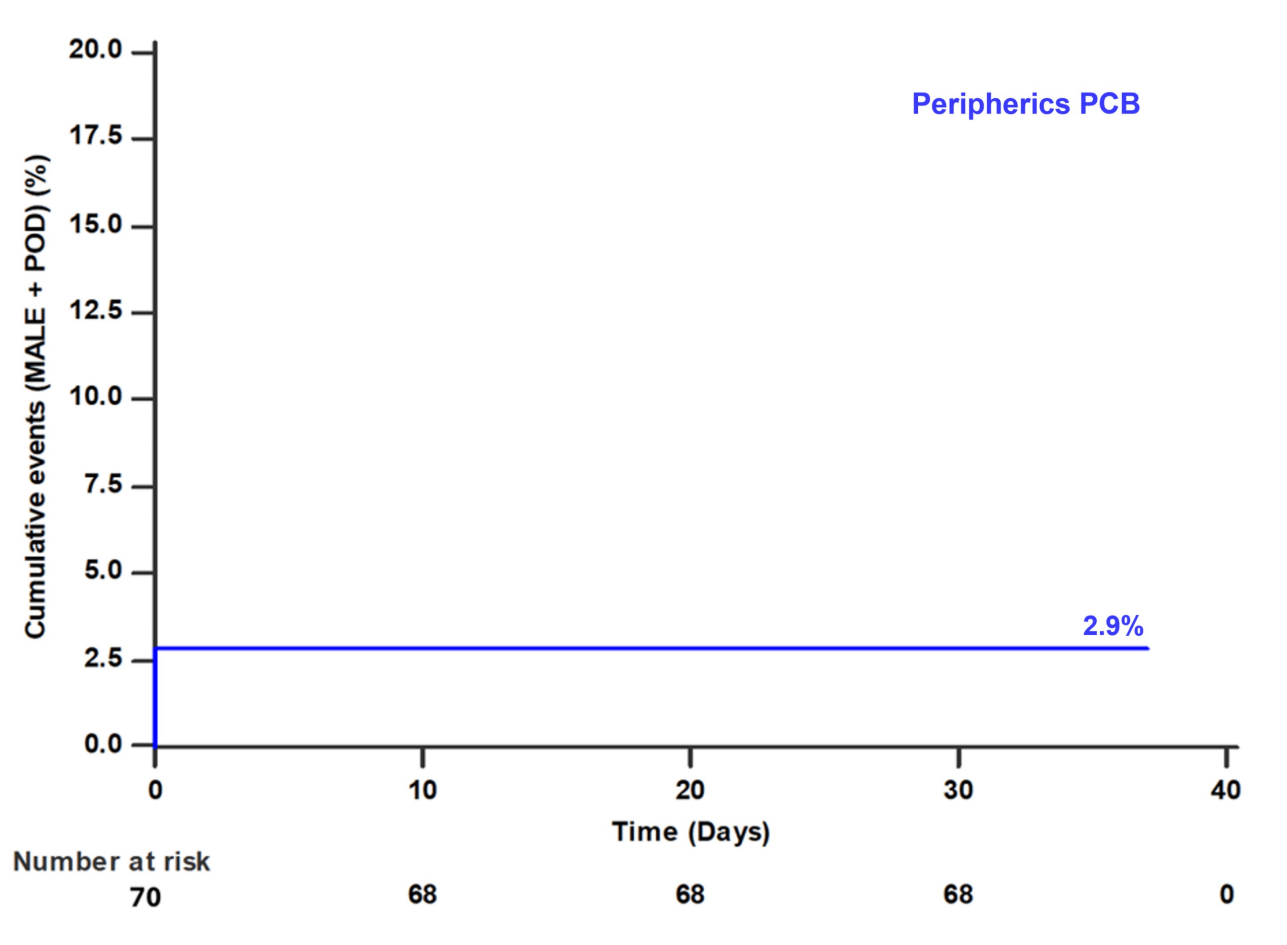
Kaplan-Meier estimate of the primary endpoint (MALE + POD) up to 30 days MALE: major adverse limb events; POD: perioperative all-cause death; PCB: paclitaxel-coated balloon

At the 30-day follow-up, the primary safety endpoint occurred in 2.9% (n=2) of patients, driven by one patient who initially underwent thrombolysis and subsequently required major reintervention (1.4%) and by one perioperative death (1.4%) attributable to sepsis during the index hospitalization in the presence of multiple comorbidities. At 30 days, MAE occurred in 2.9% (n=2) of patients, comprising one all-cause death (1.4%) and one CD-TLR (1.4%). Clinical outcomes at 30 days following the index procedure are summarized in Table 3. Substantial clinical improvement was noted at the 30-day follow-up. A total of 60 (87%) patients demonstrated at least a one-class improvement in Rutherford classification (Figure 1). At the 30-day follow-up, 58% (n=40) of patients were classified as Rutherford 0, indicating the resolution of ischemic symptoms. A significant improvement in perfusion was demonstrated, with ABI increasing from 0.51±0.15 at baseline to 0.79±0.16 at follow-up, as shown in Figure 2. Figure 3 illustrates the Kaplan-Meier estimate of the primary endpoint (MALE + POD) up to 30 days.

**Table 3:** Thirty-day clinical outcomes following the index procedure Data are represented as mean±SD or n (%). ABI: ankle-brachial index

Clinical outcomes at 30 days	Patients (N=70)
Primary endpoints	
Major adverse limb events + perioperative all-cause death	2 (2.9)
Above-ankle amputation	0 (0)
Major reintervention	1 (1.4)
New bypass graft, jump/interposition graft revision	0 (0)
Thrombolysis	1 (1.4)
Perioperative all-cause death	1 (1.4)
Secondary endpoints	
Major adverse events	2 (2.9)
All-cause mortality	1 (1.4)
Major amputation	0 (0)
Clinically driven target lesion revascularization	1 (1.4)
Rutherford class	
0	40 (58)
I	13 (18.8)
II	6 (8.7)
III	0 (0)
IV	4 (5.8)
V	6 (8.7)
Improvement in at least one Rutherford class compared to the pre-procedure	60 (87)
ABI	0.79±0.16
Change in ABI compared to the pre-procedure	0.28±0.01

## Discussion

In this study, we evaluated the clinical safety and performance of the Peripherics™ PCB catheter in patients with BTK arterial disease. The 30‐day results demonstrated encouraging early safety with several key findings: (i) the rate of MALE including POD was low at 2.9%, (ii) procedural and technical success rates were high (100% and 97.1%, respectively), and (iii) early functional improvement was observed, as shown by an increase in mean ABI from 0.51 to 0.79, with 87% of patients demonstrating at least one-class improvement in the Rutherford classification.

Many prior PCB studies for BTK angioplasty have been conducted, but only a limited number of studies have reported 30-day outcomes, which makes our study particularly valuable. At the 30-day follow-up, the AcoArt II-BTK trial [9] reported repeat angioplasty in one patient (1.6%). Similarly, in the Lutonix® 014 DCB Global BTK Registry [10] (Lutonix® 014 DCB catheter, Lutonix, Minneapolis, Minnesota, United States), the 30-day CD-TLR rate was 1.4%, and the composite MALE + POD rate was 1.4%, defined as all-cause death, above-ankle amputation, or major reintervention. In comparison, our study demonstrated an identical CD-TLR rate of 1.4%, but a slightly higher MALE + POD rate of 2.9%, which may be related to differences in lesion complexity, small-sample variability, and the tendency for Indian patients to present later in their disease course, often with more advanced ischemia and delayed care pathways.

For patients with critical limb ischemia, the first month after revascularization represents the early post-revascularization phase. Early restoration of perfusion is essential for wound stabilization, preventing tissue loss progression, and reducing infection risk [11]. Although wound-healing metrics were not systematically collected in our study, the significant improvement in ABI and Rutherford class within 30 days suggests that the Peripherics™ PCB catheter may support rapid perfusion recovery. Overall, the safety profile remains comparable, particularly given the higher-risk baseline profile of our population, characterized by older age and a greater burden of diabetes mellitus, hypertension, dyslipidemia, and more advanced ischemia (Rutherford class ≥IV). Additionally, lesion complexity was greater than in many prior registries, with total occlusions in 35.7% and calcification in 40% of treated segments [12-14].

Published data on PCB use for BTK angioplasty have shown heterogeneous outcomes, largely driven by differences in device design [12,15,16]. In a meta-analysis of eight randomized trials, PCB angioplasty was associated with lower 12-month AFS compared with plain balloon angioplasty, an effect largely attributable to the IN.PACT DEEP trial (IN.PACT Amphirion, Medtronic, Dublin, Ireland), where a manually applied urea-based coating resulted in distal paclitaxel embolization, uneven drug distribution, and drug loss during delivery [15,17]. In contrast, the Peripherics™ PCB catheter used in our study incorporates a proprietary semi-crystalline paclitaxel coating that is uniformly distributed and free of urea excipient, thereby reducing particulate loss, enhancing controlled drug transfer, and minimizing embolic risk. These design characteristics may account for the favorable early safety and performance observed in our population and are consistent with editorial commentary suggesting that earlier negative PCB outcomes were predominantly device-related and that newer-generation PCB may offer meaningful clinical benefit in BTK intervention [18]. New-generation balloons in recent studies and in our study have a reduced crystalline structure with a lower paclitaxel concentration of 2 µg/mm^2^ [19].

Although our study did not include a plain old balloon angioplasty (POBA) control arm, the low 30-day TLR and absence of major complications suggest advantages over traditional POBA, which is limited by residual stenosis, recoil, and early restenosis [20]. In addition, unlike drug-eluting stent (DES) or atherectomy BTK, both of which increase procedural complexity and are associated with higher periprocedural complication rates, DCB angioplasty offers a less complex "leave-nothing-behind" strategy that avoids permanent implants while still delivering an antiproliferative effect.

The strong deliverability profile reflected by 100% device success is clinically meaningful even in heavily calcified lesions (40% of cases). Procedural complications were infrequent and limited to flow-limiting dissection (2.8%), thrombosis (1.4%), and post-procedure occlusion (1.4%), with no instances of distal embolization or bleeding. The maintenance of perfusion of artery flow 3 in 94.3% of cases post-procedure demonstrates effective luminal restoration even in complex BTK anatomy. While the short-term results are reassuring, the Peripherics™ PCB catheter performance in BTK arteries is heavily dependent on late lumen loss and long-term patency, which require extended follow-up intervals. In this study, ongoing follow-up up to six months will provide essential data to determine sustained safety.

Limitations

This study has some limitations that should be acknowledged. First, as an observational analysis without a randomized comparator arm, it is subject to inherent selection bias and limits the ability to directly compare outcomes with other BTK revascularization strategies. Second, the sample size was modest, which may limit the generalizability of the findings. Third, because of the retrospective design, angiographic and clinical assessments were derived from routine clinical documentation rather than standardized protocols, and intravascular imaging was not routinely used in standard care, limiting the granularity of lesion morphology assessment and the precision of treatment effect evaluation. Additionally, the retrospective study design limited the availability of complete wound-healing data, preventing a systematic assessment of wound progression and healing outcomes.

## Conclusions

The Peripherics™ PCB catheter demonstrated favorable safety and clinical performance in the treatment of BTK arteries, with low event rates and marked clinical improvement at 30 days. Among the patients with BTK arterial disease and critical limb ischemia, treatment with the Peripherics™ PCB was associated with reliable procedural performance and early clinical stabilization. These findings support the integration of the Peripherics™ PCB catheter angioplasty into contemporary BTK treatment algorithms, particularly for patients in whom minimizing procedural trauma and avoiding stent implantation are clinical priorities.

## References

[1] Conte MS, Bradbury AW, Kolh P (2019). Global vascular guidelines on the management of chronic limb-threatening ischemia. Eur J Vasc Endovasc Surg.

[2] Wu SC, Marston W, Armstrong DG (2010). Wound care: the role of advanced wound healing technologies. J Vasc Surg.

[3] (2023). Global burden of peripheral artery disease and its risk factors, 1990-2019: a systematic analysis for the Global Burden of Disease Study 2019. Lancet Glob Health.

[4] Norgren L, Hiatt WR, Dormandy JA, Nehler MR, Harris KA, Fowkes FG (2007). Inter-society consensus for the management of peripheral arterial disease (TASC II). J Vasc Surg.

[5] Giannopoulos S, Varcoe RL, Lichtenberg M (2020). Balloon angioplasty of infrapopliteal arteries: a systematic review and proposed algorithm for optimal endovascular therapy. J Endovasc Ther.

[6] Tepe G, Zeller T, Albrecht T (2008). Local delivery of paclitaxel to inhibit restenosis during angioplasty of the leg. N Engl J Med.

[7] Rutherford RB, Baker JD, Ernst C, Johnston KW, Porter JM, Ahn S, Jones DN (1997). Recommended standards for reports dealing with lower extremity ischemia: revised version. J Vasc Surg.

[8] Aboyans V, Criqui MH, Abraham P (2012). Measurement and interpretation of the ankle-brachial index: a scientific statement from the American Heart Association. Circulation.

[9] Jia X, Zhuang B, Wang F (2021). Drug-coated balloon angioplasty compared with uncoated balloons in the treatment of infrapopliteal artery lesions (AcoArt II-BTK). J Endovasc Ther.

[10] Thieme M, Lichtenberg M, Brodmann M, Cioppa A, Scheinert D (2018). Lutonix® 014 DCB Global Below the Knee Registry Study: interim 6-month outcomes. J Cardiovasc Surg (Torino).

[11] Kawarada O, Yasuda S, Huang J, Honda Y, Fitzgerald PJ, Ishihara M, Ogawa H (2014). Contemporary infrapopliteal intervention for limb salvage and wound healing: harmonization of revascularization and wound management. Circ J.

[12] Zeller T, Baumgartner I, Scheinert D (2014). Drug-eluting balloon versus standard balloon angioplasty for infrapopliteal arterial revascularization in critical limb ischemia: 12-month results from the IN.PACT DEEP randomized trial. J Am Coll Cardiol.

[13] Mustapha JA, Brodmann M, Geraghty PJ, Saab F, Settlage RA, Jaff MR (2019). Drug-coated vs uncoated percutaneous transluminal angioplasty in infrapopliteal arteries: six-month results of the Lutonix BTK trial. J Invasive Cardiol.

[14] Kawarada O, Fujihara M, Higashimori A, Yokoi Y, Honda Y, Fitzgerald PJ (2012). Predictors of adverse clinical outcomes after successful infrapopliteal intervention. Catheter Cardiovasc Interv.

[15] Liistro F, Angioli P, Ventoruzzo G (2020). Randomized controlled trial of acotec drug-eluting balloon versus plain balloon for below-the-knee angioplasty. JACC Cardiovasc Interv.

[16] Zeller T, Beschorner U, Pilger E (2015). Paclitaxel-coated balloon in infrapopliteal arteries: 12-month results from the BIOLUX P-II randomized trial (BIOTRONIK'S-first in man study of the Passeo-18 LUX drug releasing PTA balloon catheter vs. the uncoated Passeo-18 PTA balloon catheter in subjects requiring revascularization of infrapopliteal arteries). JACC Cardiovasc Interv.

[17] Katsanos K, Spiliopoulos S, Kitrou P, Krokidis M, Paraskevopoulos I, Karnabatidis D (2020). Risk of death and amputation with use of paclitaxel-coated balloons in the infrapopliteal arteries for treatment of critical limb ischemia: a systematic review and meta-analysis of randomized controlled trials. J Vasc Interv Radiol.

[18] Laird JR, Armstrong EJ (2014). Drug-coated balloons for infrapopliteal disease: digging deep to understand the impact of a negative trial. J Am Coll Cardiol.

[19] Del Giudice C, Galloula A, Tiercelin C (2021). "Ranger BTK" a prospective single-centre cohort study on a new drug-coated balloon for below the knee lesions in patients with critical limb ischemia. Cardiovasc Intervent Radiol.

[20] Baumann F, Fust J, Engelberger RP (2014). Early recoil after balloon angioplasty of tibial artery obstructions in patients with critical limb ischemia. J Endovasc Ther.

